# The ectopic mandibular canines can start tooth formation in three different locations: a case series study based on single orthopantomograms from 47 individuals

**DOI:** 10.1007/s40368-024-00865-y

**Published:** 2024-03-19

**Authors:** P. Svanholt, M. Svanholt, J. Thomsen, I. Kjær

**Affiliations:** 1 Section of Orthodontics, Department of Odontology. Faculty of Health and Medical Sciences, University of Copenhagen, Copenhagen, Denmark; 2 Guldborgsund Municipal Clinic of Orthodontics, Nykøbing Falster, Denmark; 3 Copenhagen Municipal Clinic of Orthodontics, Copenhagen, Denmark; 4 Odense Municipal Clinic of Orthodontics Odense, Odense, Denmark; 5 Department of Odontology. Faculty of Health and Medical Sciences, University of Copenhagen, Copenhagen, Denmark

**Keywords:** Eruption, Ectopia, Orthopantomograms, Permanent canine, Primary canine, Mandible

## Abstract

**Introduction:**

A former study on orthopantomograms from young children with abnormal dental development (not canine ectopia) demonstrated that the tooth bud of the mandibular canine, compared to a stable longitudinal canine axis, could be located normally, anteriorly or posteriorly, with close relation to the first premolar.

**Aim:**

The aim of the present study is to analyse on orthopantomograms if the canine axis can demonstrate where the ectopic mandibular canine started tooth formation.

**Materials:**

The material consists of orthopantomograms with ectopic mandibular canines and presence of primary mandibular canines from 47 cases (29 cases 9–21 years old and 18 cases with unknown ages). The primary canines demonstrated from minor apical resorption to more severe apical resorption.

**Methods:**

Based on canine maturity, location of the canine axes and the interrelationships between the roots of the permanent canine and first premolar, the location from where the canine started tooth formation was determined. *Canine maturity.* Maturity stage below half root length and maturity stage above half root length revealed that 11 ectopic canines had less than half root length and 36 cases more than half root length. *Canine axes*. The canine axis, through the length of the primary canines Ax, is inserted on drawings of the orthopantomograms using the tracing programme Inkscape®. *Interrelationship between roots.* By visual inspection, the distance between the canine and first premolar was designated close distance, normal distance and extended distance.

**Results:**

The results are divided into 3 groups. Group 1: The initial site of the permanent ectopic canine is located within the canine axis (6 cases). Group 2: The initial site of the permanent ectopic canine is located posterior to the canine axis (36 cases). Group 3: The initial site of the permanent ectopic canine is located anterior to the canine axis (5 cases).

**Conclusion:**

The study explained that the canine axis could divide cases of ectopic canines into three groups according to the location from where tooth formation starts. For getting closer to the pattern of the ectopic canine eruption, it is necessary to analyse series of orthopantomograms taken from the same individual over several years.

## Introduction

Ectopic mandibular permanent canines are permanent canines located in different malpositions in the mandible. These positions can be horizontal locations or different oblique positions (Gonzalez-Sanchez et al. 2007; Dalessandri et al. 2017). Why and when the canines have obtained such positions are not known. It has been argued that an initial malposition of the tooth germ might explain later malposition (Joshi 2001), but this has never been proved.

Ectopic mandibular canines are normally first diagnosed in early puberty, often unexpected. Indication for early radiography of permanent canines is not obtainable. Accordingly, longitudinal radiographic images demonstrating when and how the mandibular canines develop in an ectopic position do not exist.

### Canine axis

In a recent study on orthopantomograms from young children suffering from different dental deviations, the initial positions of the crowns of the permanent canines were described (Kjær et al. 2023). The indications for taking these radiographs were predominantly arrestment in eruption of primary molars. This study concluded that the normal position of the crown of the permanent canine was below the primary canine and that the primary and permanent crowns of the canines were located in a canine axis established through the primary canine. Previous histological and anthropological studies have proved that the morphology of the alveolus of the primary canine was different from alveolae in the neighbouring incisors. This indicates that a longitudinal or length axis through the primary canine might be a stable structure in the dentition (Kjær and Bagheri 1999; Kjær 2017).

In the recent study by Kjær et al. (2023), it was also demonstrated that ectopic permanent canine crowns in the initial stages could be located in a distal (posterior) malposition, close to the anlage of the first premolar or in a mesial (anterior) malposition compared to the vertical canine axis. The malpositioned crowns were often tilted. The question not answered in this investigation by (Kjær et al. 2023) was whether the canine axis could explain the original locations of the canines which later developed into an ectopic position.

### Eruption of the permanent canine

After the permanent tooth bud has been laid down, it is well known that the crown formation will continue and later the root will develop.

**Fig. 1:**
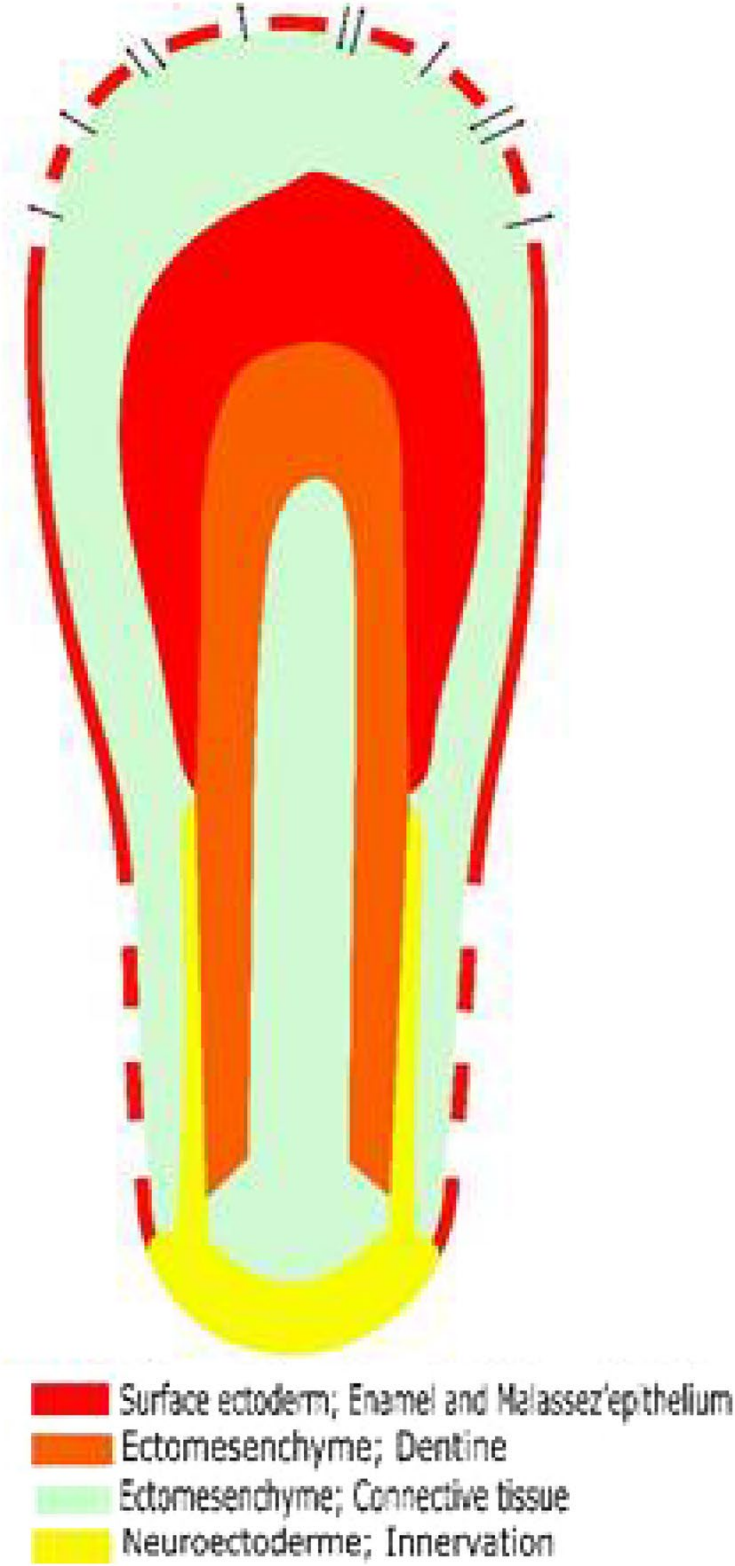
Schematic drawing indicating five types of tissues in a developing permanent mandibular canine where only half of the root has formed. The different types of tissues are marked in brackets and indicated by colours. The crown follicle in the upper part of the figure is perforated and arrows indicate cell transport between the inner part of the follicle and the overlying hard tissue. This cell transport is essential for resorbing the overlying tissue, creating a pathway for eruption. The root membrane is indicated by nervous tissue and the periodontal membrane is indicated by three tissue types, the root—close innervation layer, covered by a connective fibre layer and the outer layer of Malassez epithelium

The normal eruption process is regulated by a coordination of activity in the root membrane, the periodontal membrane and the crown follicle as described by Kjær (2014). The eruption movements seem to start when the root has obtained about half length. A schematic drawing indicating the root membrane, the periodontal membrane and the crown of a permanent canine where half of the root was formed is illustrated in Fig. 1.

It is interesting to note that a tooth always erupts in the direction of the crown because the crown follicle has the ability to break down the overlying bone and dentine, necessary for creating the eruption path (Fig. 1). The root membrane highly innervated creates the eruptive forces but does not have the ability to break down bone (Fig. 1).

During the eruptive movements, bone tissue is laid down apically. This means that the permanent tooth always moves from the initial site, where the tooth bud started dentine and enamel formation. During eruption, the tooth moves “upwards” in the direction of the crown if obstacles are not present in the eruption path, while the tooth can never move “backwards” in the direction of the root membrane. Based on insight in the normal eruption process, abnormal eruption of the mandibular premolars has been described (Kjær 2021).

Concerning canine eruption, the permanent mandibular crown and the coronal part of the root in the initial stages of the root development have a stable position close to the initial site, where dentine and enamel formation started. In later maturation stages, the root continues formation and the tooth erupts and moves from the initial site in the growing jawbone if space allows. The lower part of the root, developed in the last stages of root development, might stay at the initial site during eruptive movements of the crown.

### Hypotheses

For an ectopic permanent mandibular canine, it is presumed that the initial tooth bud, tilted or not, could have obtained a position in the region of the canine axis during eruption, but also be misplaced, compared to the canine axis, in a posterior position close to the first premolar. It is also presumed that the tooth bud compared to the canine axis could be misplaced during eruption in an anterior position.

### Aim

The aim is to analyse the location from where the ectopic mandibular canine started tooth formation. According to this aim, the permanent canine maturity was analysed and compared to the location of the canine axis and to the location of the first premolar.

## Materials and methods

### Material

The material consists of orthopantomograms with one or two ectopic mandibular permanent canines from 47 cases. The ages were unknown in 18 cases. In the remaining 29 cases, the ages were between 9 and 21 years. In all cases included in this study, the proceeding primary canines were present. In each case, the most severe ectopic canine was evaluated and designated *the ectopic canine*. In cases where the ectopic canine occurred in the left side, the orthopantomogram was flipped, so all cases studied appeared in the right side.

In 47 cases, the primary canine in the ectopic side demonstrated from minor apical resorption to more severe apical resorption.

All orthopantomograms were forwarded from orthodontic colleagues in Denmark and the Nordic countries over a period of 24 years (1998–2022). The colleagues forwarding this material raised questions on aetiology and treatment suggestions.

In one case, demonstrating bilateral ectopic canines, a colleague supplemented the orthopantomogram with a clinical photo demonstrating the ectopic canines after having penetrated the gingiva.

### Methods

#### Canine maturity on orthopantomograms

**Fig. 2:**
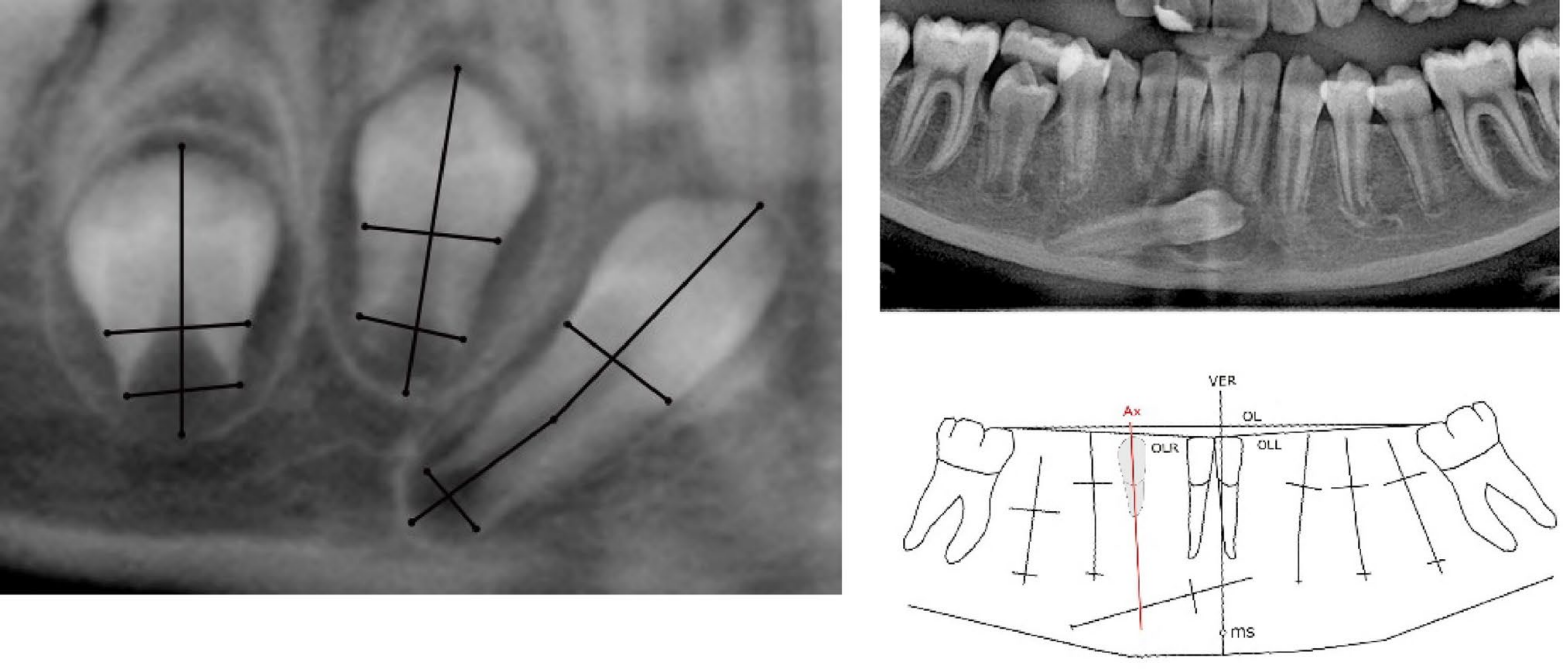
Sections of orthopantomograms indicating root maturity of permanent mandibular canine and premolars (left) and ectopic permanent mandibular canine position compared to the red vertical canine axis through the primary mandibular canine (right) Left. This figure illustrates how root development is expressed. Tooth 43 is drawn slightly angulated, showing that angulation can be seen even if it is not common. The permanent mandibular second premolar (left tooth) has just started root formation. The upper horizontal line indicates the collum-line, which marks the borderline between the crown and root. The lower horizontal line indicates the most apical part of the root dentine. The maturity in this root is less than half root length. The horizontal lines in the mandibular second premolar in the centre of the figure indicates that the root has attained half root length, while the permanent mandibular canine (right in the figure) has gained more than half root length. Right. This figure is a section of an orthopantomogram from a case, aged 13 years and 9 months, illustrating mandibular teeth between the first permanent molars (upper part of the figure) and a drawing of the same section (lower). The right permanent mandibular canine is ectopically located on the radiograph. On the drawing, the primary canine is hatched and the vertical canine axis in the primary canine is marked in red. The length axis in the premolars and canines and the horizontal lines in these teeth, explained in the left figure are marked. The figure demonstrates that the canine axis cut through the middle of the root of the ectopic canine

The maturity of the ectopic permanent mandibular canines was expressed as: maturity stage below half root length and maturity stage above half root length. This registration revealed that 11 ectopic canines had less than half root length and 36 cases had attained more than half root length. On orthopantomograms, the borderline between the crown and root is indicated by a horizontal line. Also, the positions of the lower borders of the root dentine and the root membrane are indicated by horizontal lines, as illustrated on two mandibular premolars and one canine in Fig. 2 left. These lines illustrate the maturity of the canines.

#### Canine axes on orthopantomograms

A section of an orthopantomogram including the lower border of the mandible with all teeth present between the right and the left first permanent molars is illustrated in Fig. 2 (upper right). A schematic drawing of this section is demonstrated in Fig. 2 (lower right).

The schematic drawings of the orthopantomograms are made using View Box 3® (Demetrios Halaznetis, Athens, Greece). 66 points are digitised on the section and used for tracing permanent canines and premolars (Fig. 2, lower right). Graphics of the first molars and the central mandibular incisors, the contour of the mandible, the occlusal line (OL) between the mesio-facial cusps of the right and left first molars are marked (OLR and OLL). Furthermore, the incisal edges of the central incisors and the vertical inter incisal line (VER) perpendicular line to OL line through the mental spine (ms) are inserted in the drawing.

On the orthopantomograms, the primary canines are hatched as demonstrated in Fig. 2 (lower right). Furthermore, the canine axis, through the hatched primary canines Ax (red), is inserted with the free available tracing programme Inkscape®.

#### Interrelation between roots of the permanent canines and first premolars

By visual inspection, the interrelation between the permanent canine and first premolar roots was expressed as: Normal distance, close distance or extended distance.

#### Analysis of where the ectopic canine started tooth formation

##### Canine maturity, less than half rooted

Permanent ectopic canines, half rooted or less than half rooted, stay at the location where the tooth bud was laid down. The canine axis determines if the location can be considered a normal location within the canine axis, a posterior position, close to the first premolar, or if the position is a mesial position.

##### Canine maturity, more than half rooted

In cases where the root was more than half-sized, the comparison of the permanent canine location to the canine axis, and to the distance of the apical part of the first premolar, determined the initial location, analysed as follows.

1. If the canine axis cuts through the crown of the permanent canine, and/or the apical part of the root, the original location of the tooth bud is within the canine axis (normal).
2. If the canine axis cuts through the root, located close to the first premolar, the original tooth bud has a posterior location.
3. If the canine axis does not cut the ectopic canine at all, and the distance to the first premolar is extended, then the original location of the tooth bud is mesial.

## Results

The results are divided according to the original location of the permanent canine compared to the stable canine axis and to the lower part of the first premolar.

This comparison resulted in 3 groups illustrated below.

**Fig. 3:**
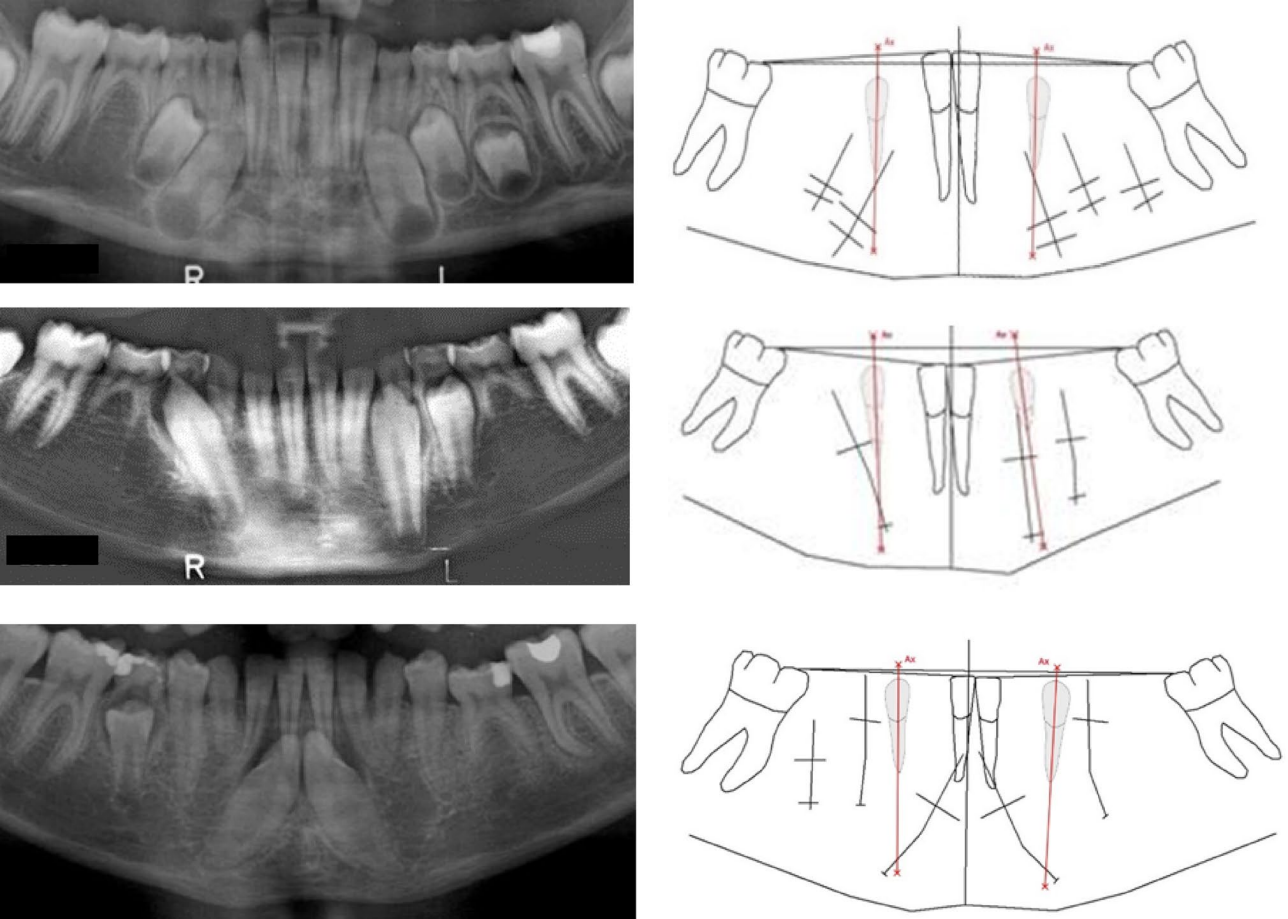
Sections of three orthopantomograms presents the mandibular teeth demonstrated to the left. From above, the orthopantomograms are from cases, 9 years old, 19 years old and a case with unknown age). Drawings corresponding to each radiograph (introduced and explained in Fig. 2) with inserted canine axis appear to the right. The canine axis cut the permanent canine crown or the apex of the canine. This might indicate that the mandibular canine started tooth formation in normal locations below the primary canines but that the positions of the tooth buds seemingly were tilted initially

*Group 1* In this group, the initial site of the permanent canine is located within the area of the canine axis. Six cases were registered in this group and three are illustrated in Fig. 3. One of the three cases had a short root length and the canine axis cut the crown of the permanent canine. In the two other cases, the canine axis cut the apical part of the permanent canine in a region not close to the first premolar.

**Fig. 4:**
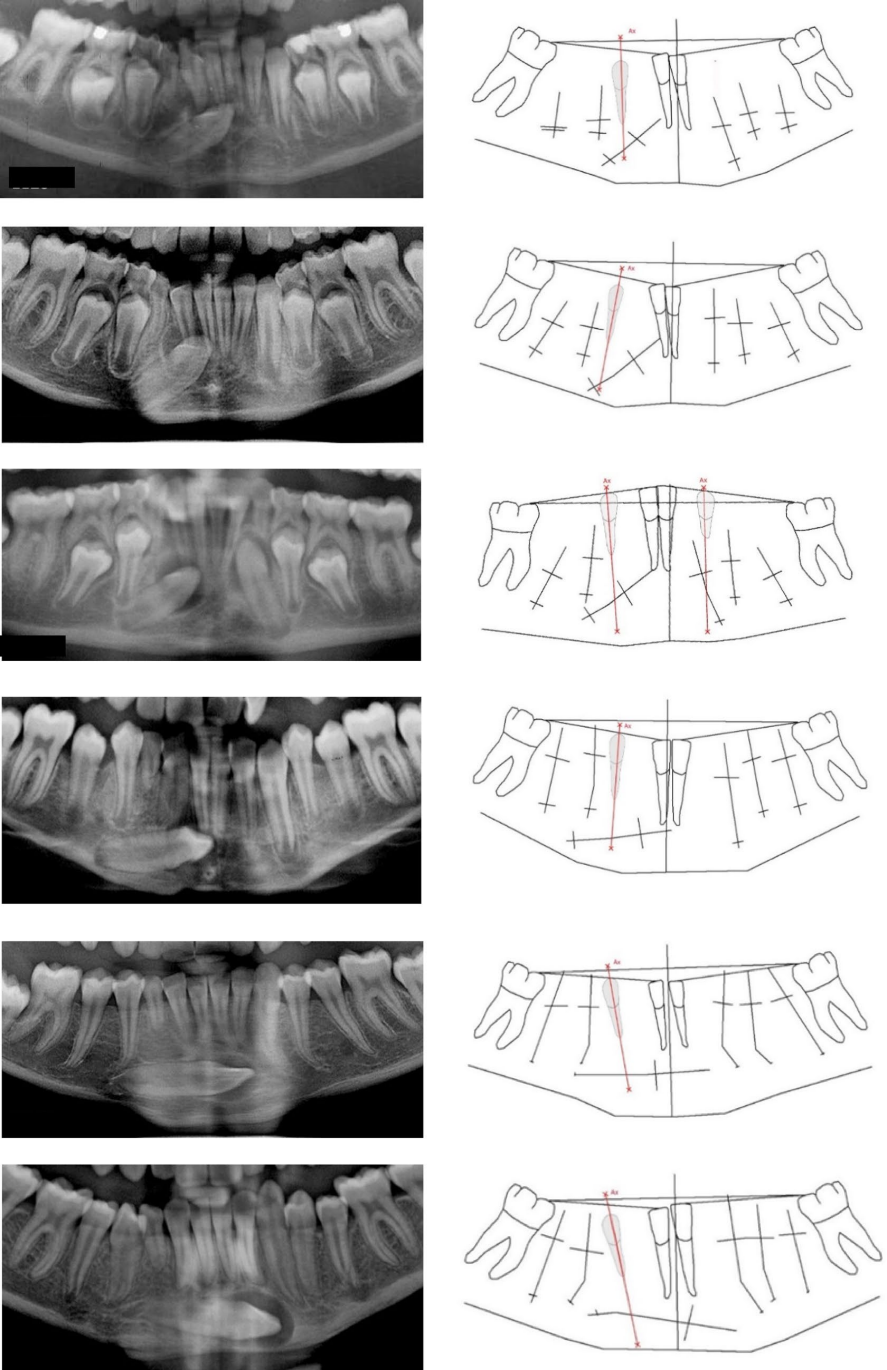
Sections of six orthopantomograms presenting the mandibular teeth are demonstrated to the left (from above, the orthopantomograms are from cases, 10 years old, 11 year and 3 months old, 10 year and 10 months old, 12 year and 4 months old, a case with unknown age and a case 11 years old). Drawings of the radiographs (introduced and explained in Fig. 2) with inserted canine axis appear to the right. The canine axis cuts the newly formed lower part of the permanent canine root. This indicates that the mandibular canines started tooth formation in posterior locations seemingly tilted initially. The maturity of the ectopic mandibular canines increases from the upper case to the two lower cases, where the canine root is fully developed

*Group 2* The initial site of the permanent canine is located posterior to the canine axis. In these cases, the canine axis did not cut the crown of the permanent canine and there was a very short distance between the roots of the canine and first premolars. 36 cases were registered in this group and of these, six cases are demonstrated in Fig. 4.

**Fig. 5:**
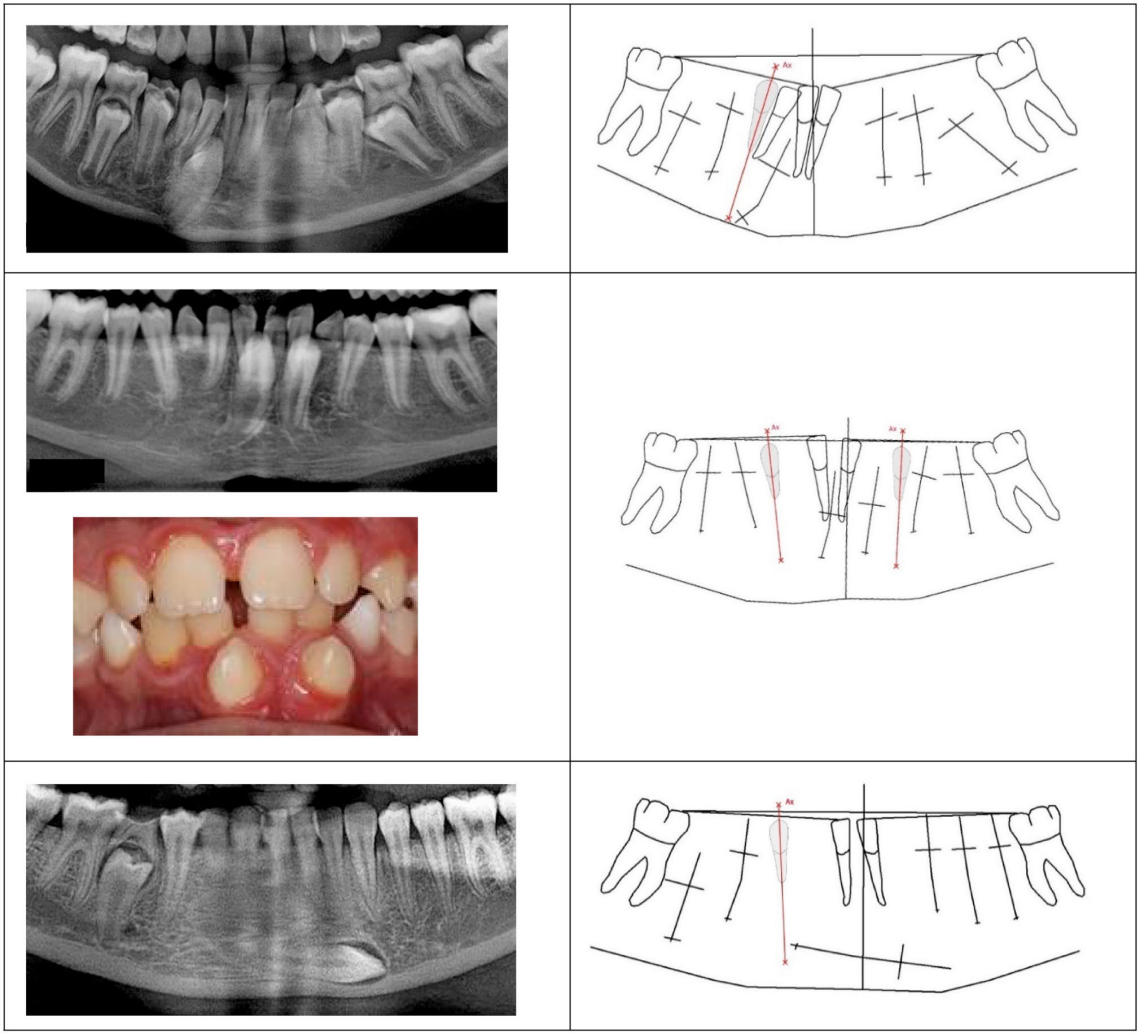
Sections of three orthopantomograms presenting the mandibular teeth are demonstrated to the left (from above, the orthopantomograms are from cases, 10 year and 9 months old, 12 year and 3 months old and 16 years old). Drawings of the radiographs (introduced and explained in Fig. 2) with inserted canine axis appear to the right. The canine axis does not cut the permanent canine root. This indicates that the mandibular canines started tooth formation anteriorly to the normal location. The mandibular canine in the upper case is less mature than in the other cases. The clinical photo illustrates eruption of the ectopic canines. In the last case demonstrated, it cannot be determined if the original site of the ectopic canine was posterior or anterior to the canine axis

*Group 3* The initial site of the permanent canine is located anterior to the canine axis. Five cases were registered in this group. In one case, the exact location for onset of the permanent crown formation could not be determined. This case and two cases belonging to group 3 are demonstrated in Fig. 5.

## Discussion

The ectopic canines have started tooth formation seemingly in a tilted position either within the canine axis (Group 1), posteriorly to the canine axis (Group 2) or anterior to the canine axis (Group 3). The last case shown in Fig. 5 is an example of this uncertainty. The mandibular canine in this case seems to have moved a long distance during eruption and could as well have been originally positioned posteriorly as anteriorly to the canine axis.

Ectopic eruption of the permanent mandibular canine appears as a mystery, which over the years has been discussed regularly (Mapparapu 2002; Delessandri et al. 2017; Batwa and Alzain 2018). The main problem has been a lack of longitudinal studies, answering the questions why is the canine ectopic? Can it be prevented, predicted and treated?

The ectopic mandibular canine has often been studied in cross-sectional studies and the focus has often been: where did the permanent canine come from? Is the primary mandibular canine present, resorbed or not in these cases? Few cases are dealing with surgical treatment (Cavoti et al. 2016). A recent radiographic study on mandibular canine eruption demonstrated that the canine crown morphology appeared different on different orthopantomograms, with canines located in different positions (Svanholt et al. 2020). The canine morphology could either be designated “facial morphology” as the morphology observed in the facial view or “lateral morphology” as observed in the lateral view. Facial morphology of the crown seemed to appear in cases where eruption was close to normal, while lateral morphology appeared in more complicated, often horizontally located, canine cases (Svanholt et al. 2020). This former observation seems to be confirmed in the present study, where facial morphology is registered in Figs. 2 and 3 (upper and middle cases), while lateral view is predominantly seen in Figs. 4 and 5 (lower case). Insecurity and errors might be noticed in orthopantomographic studies.

Another aspect in studies of eruptive movements is the simultaneous jaw growth. By comparing radiographs of the same mandibular canine under eruption, it may appear that the canine moves downwards during the eruption, but this could be an optical illusion, caused by alveolar bone growth.

The mandibular growth has been studied in prenatal specimens and in children. It was demonstrated in prenatal life, that the mandibular bone formation started in the canine region (Kjær 1975, 1989, 1990). In these studies, also formation of the primary canine appeared. In the study of Kjær and Bagheri (1999), specific focus was on the alveolar bone, surrounding the mandibular canine. In this study, it was demonstrated that the alveolus of the canine was not ossified in the facial aspect, which was not the case in the neighbouring incisors. This gave the impression that the canine was a stabile tooth, not moving mesially or distally during development. Also, the mental foramen seems to have a stable position, only dependent on changes as a result of facial bone apposition (Kjær 1989). These prenatal studies may indicate canine and bone stability in the actual region, but it is difficult to prove this and impossible to follow up in longitudinal studies.

Former studies on ectopic mandibular canines have also focused on the resorption aspect of the roots of the primary canines. It has been demonstrated that resorption occurs in the primary canines without pressure from erupting permanent canines. This is also proved in the present study. Cases where the primary canines were extracted or totally resorbed and exfoliated were not included in this study.

The present study raises the question whether the crown follicle has an inborn “mechanism” steering the eruption direction. It is supposed that the tilting of the early tooth bud could result in abnormal function of the crown follicle. This is a hypothesis, which cannot be proved in the present study.

The usability of an orthopantomogram in the visual analysis of bone morphology and root morphology is also dubious because the orientation of the patient is a main factor for obtaining valuable and comparable results. Another aspect of importance is that the exact time for onset of eruptive movements of a tooth is unknown. Is it when the root has gained half length or possibly before? In this connection it is also problematic that the root is estimated as “half-sized”, when the final root length is not known. Furthermore, it is questioned if the teeth always erupt at the same stage of root maturity in different individuals.

Developmental stages of permanent canines have been studied by Svanholt and Kjær (2008), and eruption times of permanent canines have been investigated by Parner et al. (2002). The agreement in maturation and eruption times in these two studies is convincing for at study like the present.

The present cross-sectional study, with all the questions raised, gives a new insight in different locations from where the ectopic mandibular canines have started tooth formation. Instead of many cross-sectional studies from prenatal and postnatal periods, it would be extremely valuable to obtain results from postnatal longitudinal studies of ectopically erupting mandibular canines.

## Conclusion

The study explained that the canine axis could divide cases of ectopic canines into three groups according to the location from where tooth formation starts. For getting closer to the pattern of the ectopic canine eruption, it is necessary to analyse series of orthopantomograms taken from the same individual over several years.

## Data Availability

Due to the nature of the research, supporting data is not available.
